# Task‐related brain activation moderates age‐related structural decline in cognitive flexibility: A longitudinal study

**DOI:** 10.1002/alz.092613

**Published:** 2025-01-03

**Authors:** Fatemeh Hasanzadeh, Yaakov Stern, Christian G Habeck, Elaine Gazes

**Affiliations:** ^1^ Columbia University Irving Medical Center, New York, NY USA; ^2^ Taub Institute for Research in Alzheimer’s Disease and the Aging Brain, Columbia University, New York, NY USA; ^3^ Columbia University Vagelos College of Physicians and Surgeons, New York, NY USA

## Abstract

**Background:**

Aging is accompanied by declines in brain structure and executive functions like cognitive flexibility ‐ the ability to switch between tasks. However, there are substantial individual differences in degree of impairment, as some elders maintain high functioning despite accumulation of brain damage. This variability suggests higher cognitive reserve (CR) which enables older adults to cope more effectively with structural brain changes. We examined whether greater task‐related brain activation can mitigate effects of structural decline on flexibility over time, operating as a neural CR mechanism.

**Methods:**

82 older and 21 young adults completed MRI scans and a set‐shifting paradigm with switch and nonswitch conditions at baseline; 75 older and 17 younger returned after 5 years. Switch cost, measured as the reaction time difference between switch and nonswitch conditions, indicating flexibility. We integrated cortical volume, cortical thickness, white matter hyperintensity burden, fractional anisotropy and mean diffusivity into a multivariate structural index using an autoencoder technique. We applied ordinal trend analysis to baseline fMRI data to identify patterns displaying increases in activation from nonswitch to switch conditions. The difference in pattern expression scores between conditions indicated differential task‐related activation (dOrT). We tested if baseline dOrT moderates the impact of structural changes on 5‐year switch cost changes using a linear mixed effect model.

**Results:**

The results indicated a positive main effect of brain structural index (β = 0.129, p<0.05) and a negative interaction term of brain index and dOrT (β = ‐0.282, p<0.05) on switch cost in older adults. The significant interaction demonstrates that with higher dOrT, brain structural index related less strongly to rising costs. This moderation effect implies enhanced engagement of specified OrT patterns during demanding conditions buffers effects of age‐related structural deterioration on declining cognitive flexibility.

**Conclusions:**

Older adults utilizing task‐related functional brain networks to meet increasing demands showed attenuation of effects of structural decline on reducing cognitive flexibility over time. Results suggest differential activation of identified functional networks, serving as a neural implementation of CR, elucidating neuroprotective factors that preserve executive functions into advanced age.

